# Characteristic Aroma Compounds from Different Pineapple Parts 

**DOI:** 10.3390/molecules16065104

**Published:** 2011-06-20

**Authors:** Chang-Bin Wei, Sheng-Hui Liu, Yu-Ge Liu, Ling-Ling Lv, Wen-Xiu Yang, Guang-Ming Sun

**Affiliations:** 1 South Subtropical Crop Research Institute, Chinese Academy of Tropical Agricultural Science, Guangdong, Zhanjiang, 524091, China; 2 Yunnan Vocational College of Tropical Crops, Yunnan, Pu’er, 665000, China

**Keywords:** pineapple, pulp, core, characteristic aroma compounds

## Abstract

Characteristic aroma volatile compounds from different parts of cayenne pineapple were analyzed by headspace-solid phase microextraction (HS-SPME) and gas chromatography-mass spectrometry (GC/MS). The main volatile compounds were esters, terpenes, ketones and aldehydes. The number and content of aroma compounds detected in pulp were higher than those found in core. In pulp, the characteristic aroma compounds were ethyl 2-methylbutanoate, ethyl hexanoate, 2,5-dimethyl-4-hydroxy-3(2*H*)-furanone (DMHF), decanal, ethyl 3-(methylthio)propionate, ethyl butanoate, and ethyl (*E*)-3-hexenoate; while in core the main compounds were ethyl 2-methylbutanoate, ethyl hexanoate and DMHF. The highest odor units were found to correspond to ethyl 2-methylbutanoate, followed by ethyl hexanoate and DMHF. The odor units found for pulp were higher than those for core.

## 1. Introduction

Pineapple, named ‘the king of fruit’ because of its crown of leaves, is now produced throughout the tropical and subtropical areas, representing an important horticultural industry. Due to its attractive sweet flavor, pineapple is widely consumed as a fresh and canned fruit, as well as in processed juices and as an ingredient in exotic foods [1]. The volatiles of pineapple have been studied for over 60 years, and more than 280 aroma compounds have been identified to date [1,2]. 

The aroma volatile compounds are important properties of fruits, and are vital factors to determine the attributes of fresh and processed fruit. Currently there is an increasing interest in research related to the aroma volatiles of fruits all around the World, especially in China [3,4,5,6]. For pineapple, volatile compounds of different varieties [7,8], different crop areas [9], different ripening stages [10], fruit development [11,12], storage conditions [12] and flesh position of Gold cultivar [13] (top, middle, and bottom cross-sections along the central axis of the fruit) have been studied recently, but no research work has been done on the aroma of pulp and core of the fruit. 

Solid-phase microextraction (SPME) is a solvent-free, rapid and sensitive technique which has become popular in volatile flavour analysis [14], now widely used for analysis of aroma volatiles in many food and beverage matrices [15], due to its simplicity of manipulation. This method has been successfully used for qualitative and quantitative analysis of volatile compounds in various fruits [16,17,18].

It is very significant for fruit quality, selection and breeding, cultivation as well as industrial development to study characteristic attributes of pineapple aroma. The availability of detailed information about differences among the aroma volatile compounds between pulp and core of pineapple was limited. The main objective of this study was to investigate the volatiles and odor activity values from pineapple cultivated in Yunnan province using HS-SPME-GC-MS. 

## 2. Results

### 2.1. Volatile Compounds in Different Parts of Pineapple Fruit

Table 1 shows the volatile compounds observed in pulp and core of Smooth Cayenne pineapple. The identification was accomplished by comparison of their MS spectra with those present in a reference data library (NIST2005). The analysis of pulp and core displayed the presence of 44 volatile compounds, of which 18 were esters, 17 were terpenes, and four were alkenes.

**Table 1:** Aroma volatile compounds in pineapple pulp and core.

Category	Compound name ^a^	Content (µg·kg^−1^)	
		pulp	core
Esters	Ethyl butanoate	6.09	-
	Ethyl 2-methylbutanoate	10.16	-
	Methyl hexanoate	24.96	7.55
	Ethyl hexanoate	106.21	48.42
	Ethyl (*E*)-3-hexenoate	5.81	-
	Methyl 3-(methylthio)propanoate	27.38	10.65
	2,3-Butanediol diacetate	4.55	4.10
	Ethyl 3-(methylthio)propanoate	91.21	42.67
	Ethyl 3-acetoxybutyrate	3.68	3.03
	Methyl 4-octenoate	2.23	-
	Methyl octanoate	8.39	3.19
	Ethyl 3-hydroxy hexanoate	23.55	19.84
	Diethyl succinate	1.17	0.88
	Ethyl 4-octenoate	18.34	12.24
	Ethyl nonanoate	59.88	-
	Ethyl phenylacetate	1.98	3.31
	Methyl decanoate	1.02	-
	Ethyl decanoate	11.11	3.67
Terpenes	α-Terpineol	-	1.54
	(+)-Sativene	2.35	-
	β-Caryophyllene	-	2.58
	(+)-Cycloisosativene	1.55	2.28
	α-Cubebene	11.0	-
	Copaene	-	19.16
	β-Elemene	2.98	3.92
	β-Cubebene	0.65	-
	(+)-Calarene	-	1.26
	γ-Amorphene	10.87	19.65
	β-Selinene	3.66	-
	α-Muurolene	22.23	28.94
	β-Guaiene	0.58	-
	Valencene	-	1.10
	(+)-δ-Cadinene	4.91	7.27
	α-Calacorene	0.99	1.32
	(*Z*)-β-Ocimene	1.44	-
Lactones	γ-Hexanolactone	2.76	2.61
Ketones	DMHF	3.19	-
	2,10,10-Trimethyltricyclo[7.1.1.0(2,7)]undec-6-en-8-one	-	2.80
Aldehydes	Nonanal	-	3.91
	Decanal	2.94	3.20
Hydrocarbons	1,3,5,8-Undecatetraene	5.13	9.06
	Eudesma-4(14),11-diene	-	6.03
	1,2,4,8-Tetramethylbicyclo[6.3.0]undeca-2,4-diene	-	4.42
	Nonylcyclopropane	-	3.56
Total		484.95	284.16

Note: ^a^: The compounds of identification were tentative; - : Not detected.

The total concentration of aroma in pulp was 484.95 µg·kg^−1^, with ethyl hexanoate presenting the highest concentration (106.21 µg·kg^−1^), followed by ethyl 3-(methylthio)propanoate (91.21 µg·kg^−1^). 

In pineapple core, 31 volatile compounds were identified. The total concentration in the core reached 284.16 ug·kg^−1^, with ethyl hexanoate as the main compound (48.42 µg·kg^−1^), followed by ethyl 3-(methylthio)propanoate (42.67 µg·kg^−1^). 

In brief, 21 kinds of volatile compounds were found in both pineapple pulp and core, which included 11 esters, seven terpenes, one lactone, one aldehyde and one alkene.

### 2.2. Analysis of Groups and Contents of Aroma Volatile Compounds in Pineapple

The results from analysis of aroma volatiles showed esters and terpenes as the most abundant volatile compounds in both pulp and core of pineapple (Table 1), and their percentage represented 84.07% and 13.03% in pulp and 56.15% and 31.33% in core. The ester levels were 1.5 times higher in pulp than in core, whereas terpene content in pulp was lower than those in core. Esters represented 73.76% of total aroma production in pineapple fruit, which included terpenes (19.79%), lactones (0.70%), ketones (0.78%), aldehydes (1.31%) and alkenes (3.67%).

### 2.3. The Characteristic Aroma Compounds of Pineapple

In Table 2 are listed the orthonasal odor threshold values in water and odor activity values (OAV) for several volatile compounds found in pineapple. 

**Table 2:** Characteristic aroma compounds and odor units in pineapple pulp and core.

Aroma compounds	Odor threshold ^a^ (µg kg^−1^)	Odor activity value (OAV)	
		pulp	core
Methyl 3-(methylthio)propanoate	180	0.15	0.06
Ethyl 3-(methylthio)propanoate	7	13.03	6.10
Ethyl butanoate	1	6.09	-
Ethyl 2-methylbutanoate	0.006	1693.33	-
Methyl hexanoate	70	0.36	0.11
Ethyl hexanoate	0.76	139.75	63.71
Ethyl (*E*)-3-hexenoate	2	2.91	-
DMHF	0.03	106.33	-
β-Caryophyllene	64	-	0.04
Methyl octanoate	200	0.04	0.02
α-Terpineol	330	-	0.005
Nonanal	1	-	3.91
Decanal	0.1	29.4	32.0
Ethyl decanoate	6300	0.0018	5.80*10^−4^

^a^ odor thresholds reported by: Tokitomo *et al.* [2]; Feng *et al.* [3]; Jorge *et al.* [19]; Chen *et al.* [20].

Seven characteristic aroma compounds were detected in pineapple pulp (Table 2), of which, ethyl 2- methylbutanoate showed the highest odor activity value (1,693.33), followed by ethyl hexanoate, DMHF, decanal, ethyl 3-(methylthio)propanoate, ethyl butanoate and ethyl (*E*)-3-hexenoate, in decreasing order. 

Similarly, four characteristic aroma compounds were identified in pineapple core. Based on their odor activity value, ethyl hexanoate contributed to the core aroma in a greatest degree, followed by decanal, ethyl 3-(methylthio)propanoate and nonanal.

## 3. Discussion

### 3.1. The Aroma Volatile Compounds of Pineapple

More than 280 volatile compounds had been identified among the aroma volatiles of pineapple, whereas only a few volatiles contribute to pineapple aroma [2]. It has been reported that esters were the most abundant pineapple volatiles, in particular, ethyl hexanoate and methyl hexanoate which have the highest contribution to the pineapple aroma [21]. Recently, Marta *et al.* [13], Elss *et al.* [22] and Taivini *et al.* [23], Umano *et al.* [24], Akioka *et al.* [25] also reported that esters were the major volatile compounds in pineapple volatile composition, which was in agreement with our results, however, He *et al.* [26] reported that hydrocarbons and esters were the main compounds, which could be explained by differences in cultivars, growing conditions, and volatiles extraction methods. Such differences could also justify why methyl butanoate and methyl 2-methylbutanoate were not found in ‘Smooth Cayenne’ pineapple, despite being the main abundant components in other studies [13,22,23]. 

The results of Berger [27] were also different from ours, as in his work two minor hydrocarbon compounds, 1-(*E,Z*)-3,5-undecatriene and 1-(*E,Z,Z*)-3,5,8-undecatetraene were important contributors to fresh-cut pineapple aroma due to their low odor threshold values. Also the results of Takeoka *et al.* [28] were different from ours, as they identified many sulfur-containing esters among pineapple volatiles, nevertheless, their concentrations were lower than their odor thresholds. 

‘Smooth Cayenne’ pineapple is commonly used as the source of canned fruit and processed juice in China. It can be seen from the results that there were many differences between the pulp and core of pineapple, which indicated that this fruit could be comprehensively used. The aroma volatiles in pulp differed significantly from those in core in the present study. The aroma content in pulp was 1.7 times higher than that in core, thus aroma volatiles were concentrated in pulp of pineapple. As an example, it was reported that volatile compounds content varied from the top to the bottom cross-section of Gold cultivar pineapple [13]. The research on aroma volatiles in different parts of mango was also done [29], which demonstrated that most of the aroma volatile compounds seemed to be concentrated in different position of fruits. 

### 3.2. The Characteristic Aroma Compounds of Pineapple

The odor activity value was used to be described the contribution of aroma volatile compound to overall fruit flavor. When the OAVs > 1 for a compound, which would play a critical role for fruit flavor, and was thought to be the characteristic aroma compounds of fruits. The characteristic aroma compounds of different fruits were significantly different. 

Engel *et al.* [30] reported that several methyl esters and some characteristic sulphur-containing esters, various hydroxy esters and their corresponding acetoxy esters, as well as a number of lactones were responsible for the typical pineapple flavor profile. It was also reported that ethyl acetate [24], methyl butanoate [13,22], ethyl butanoate [13], 2,5-dimethyl-4-methoxy-3(2*H*)-furanone (DMMF) [13,22,25], DMHF [1,2,22,25], ethyl 2-methylbutanoate [1,2], methyl 2-methylbutanoate [22], methyl 3-(methylthio)propionate [22], ethyl 3-(methylthio)propionate [22,24], methyl hexanoate [22] and ethyl hexanoate [22] were considered to be odor active, and contribute to the overall aroma of pineapple.

In our work, the characteristic aroma compounds were mainly concentrated in pulp. Ethyl 2-methylbutanoate, ethyl hexanoate, DMHF, decanal, ethyl 3-(methylthio)propanoate, ethyl butanoate, ethyl (*E*)-3-hexenoate were the main characteristic aroma compounds in pulp; while ethyl hexanoate, decanal, ethyl 3-(methylthio)propanoate and nonanal were the main compounds in core.

## 4. Experimental

Smooth Cayenne pineapples (*Ananas comosus* L. Merr) were obtained from Xishuanbanna (Yunnan Province) at the same maturation degree. The initial quality characteristics of the pineapples are shown in Table 3.

**Table 3:** Initial pineapple quality characteristics.

Category	Content	Category	Content
firmness (Kg)	0.60 ± 0.10	a*	−3.91 ± 0.77
SSC (Brix)	12.50 ± 0.50	b*	−4.77 ± 0.71
L*	42.07 ± 2.40		

The soluble solid content (SSC) was determined using an Atago Hand Refractometer (ATAGO, master-m, Japan). The fruit firmness was measured by a hardness tester model FHM-5 (FHM, Japan). The pineapple pulp color expressed as Hunter L, a, and b values, was measured on the most and least colored parts of three fruits from each variety using a colorimeter (Tintometer, Lovibond RT100, UK). The L*data represent the whiteness of the colour with 100 representing white and 0 representing black. The a* data represent red and green: a positive value indicates a red colour with +60 being the maximum, while a negative value indicates a green colour with -60 being the maximum. Similarly, b* represents yellow and blue, (+) being yellow and (-) indicating blue [19].

The pulp and core from peeled pineapples were separated, stirred and mixed using a food processor. NaCl was added into each sample (10:2 ratio of weight sample to NaCl) and homogenized, then homogenized sample (10 g) was put into a 25 mL vial (Supelco) for extracting volatile compounds by HS-SPME. The SPME syringe (65 μm polydimethylsiloxane/divinylbenzene) was then manually inserted into the headspace of the vial and the aroma was extracted at 25 °C for 40 min with continuous stirring. The aroma compounds were identified using a gas chromatograph (GC, Agilent 6890N) coupled to a mass spectrometer (MS, Agilent 5975, USA) equipped with a HP-5MS (30 m × 0.25 mm × 0.25 μm film thickness, HP, USA) capillary column.

After the extraction, the SPME fiber was placed into the GC-MS injector and the fiber was maintained for 2 min for desorption. Temperature program was as follows: 40–120 °C at 3 °C /min, 120–200 °C at 5 °C /min, 200 °C (10 min). Injector temperature, 250 °C; Carrier gas, helium (1.0 mL/min); mass range, m/z 35 to 335 a.m.u. Identification was accomplished by comparison of the mass spectra with those in the NIST2005 library of standard compounds. Quantification was performed using octadecane as internal standard. 

The characteristic aroma compounds were defined by their odor activity values (OAVs) which were calculated by the ratio of the concentration of each component to its odor threshold [31]. The compound was assumed to contribute to characteristic aroma compounds and considered as characteristic aroma compound when its OAV > 1.

## 5. Conclusions

The main volatile compounds found in pineapple pulp and core were esters, followed by terpenes, ketones and aldehydes. The content and number of compounds detected in pulp was higher than those found in core. The characteristic aroma compounds found in pulp were ethyl 2-methylbutanoate, ethyl hexanoate, DMHF, decanal, ethyl 3-(methylthio)propanoate, ethyl butanoate, and ethyl (*E*)-3-hexenoate; while the characteristic ones in the core were ethyl 2-methylbutanoate, ethyl hexanoate and DMHF. The compound presenting the highest odor units was ethyl 2-methylbutanoate, followed by ethyl hexanoate and DMHF. In our work, the odor units in pulp were higher than those in core, suggesting the characteristic aroma profile of pineapple could be closely related to the pulp volatile composition.
